# Poly(4-amino-3-hydroxynaphthalene-1-sulfonic acid) modified glassy carbon electrode for square wave voltammetric determination of amoxicillin in four tablet brands

**DOI:** 10.1186/s13065-021-00739-0

**Published:** 2021-02-08

**Authors:** Adane Kassa, Meareg Amare

**Affiliations:** 1 Bahir Dar University, Bahir Dar, Ethiopia; 2 Debremarkos University, Debremarkos, Ethiopia

**Keywords:** Amoxicillin, Antibiotics, β-Lactam, Electropolymerization, Spike recovery, Standard addition

## Abstract

**Background:**

Amoxicillin (AMX), which is one of the β-lactam antibiotics used in the treatment of bacterial infections, is known to have a serious mechanism of resistance necessitating continuous monitoring of its level in pharmaceutical and serum samples.

**Results:**

In this study, we presented selective, accurate, and precise square wave voltammetric method based on poly(4-amino-3-hydroxynaphthalene-1-sulfonic acid) modified glassy carbon electrode (poly(AHNSA/GCE)) for determination of amoxicillin in four selected tablet brands. Appearance of a peak in the oxidative scan direction without a peak in the reductive direction of cyclic voltammograms of both bare GCE and poly(AHNSA/GCE) with four folds current and much reduced potential on the modified electrode showed catalytic property of the modifier towards oxidation of AMX. While cyclic voltammetric studies of effect of scan rate showed predominantly diffusion controlled oxidation of AMX with one electron participation, effect of pH revealed participation of protons and electrons in a 1:1 ratio. The square wave voltammetric peak current response of the modified electrode for AMX showed linear dependence on the concentration of the spiked standard AMX in the range 10–150 µmol L^−1^ with 9.9 nmol L^−1^ LOD. The AMX content of the studied tablet brands were found in the range 97.84–100.78% of the labeled value. Spike recovery results of 99.6–100.5%, and interference recovery results of 95.4–100.8% AMX in the presence of 50–200% of ampicillin and cloxicillin validated the applicability of the method for determination of amoxicillin in tablet formulation.

**Conclusion:**

In contrast to the previously reported works on determination of amoxicillin, the present method showed an excellent performance making it a potential method for determination of amoxicillin in real samples including serum samples.

## Introduction

The antibacterial action of β-lactam antibiotics reposes in the inhibition of the active site of penicillin-binding proteins (PBP) [1]. Penicillins are a group of β-lactam antibiotics which contain a β-lactam ring fused to thiazolidine ring [1, 2].

**Scheme 1:**
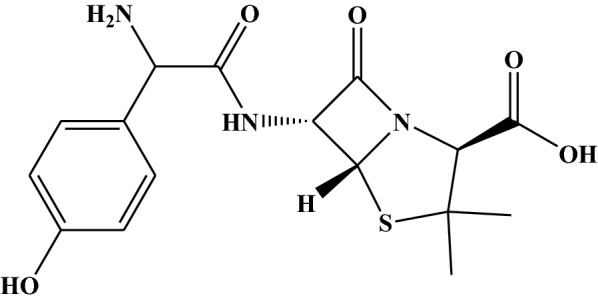
The chemical structure of amoxicillin

Amoxicillin (d-α-amino-*p*-hydroxybenzylpenicillin trihydrate) (Scheme 1), which is a β-lactam semisynthetic penicillin from the aminopenicillin class with a broad antibacterial spectrum, is used to treat a large number of infections with susceptible gram-positive and gram-negative bacteria in both human and animals [2, 3]. The fact that amoxicillin (AMX) is better absorbed following oral administration than other β-lactam antibiotics, it remained the most frequently prescribed penicillin derivatives within the class [2]. Because of its fair safety and efficacy, AMX is one of the most important antibiotics used in the treatment of bacterial infections [3, 4]. Despite a high level of clinical success, a serious mechanism of resistance had emerged demanding high dose regimen and new pharmacokinetic combination [2]. Common adverse drug reactions associated with use of AMX includes diarrhoea, stomach upset, headache, abnormal taste sense, skin rash and vaginal yeast infection [5]. Thus, monitoring the level of AMX in drugs and biological fluids has attracted the attention of researchers.

Spectrophotometric [6, 7], chromatographic [8–10], and hyphenated techniques like LC/MS/MS [11] are among the commonly reported techniques for determination of AMX. However, all these techniques are known to have disadvantages including tedious sample pre-treatment, long analysis time, expensive instrumentation, and large volume of organic solvents making them environmentally unfriendly [3, 12–14]. In contrast to the conventional methods, low cost, good biocompatibility, short response time, environmentally friendly, and high selectivity make electrochemical methods to be superior [3, 12, 15].

Attempts have been made on electrochemical determination of AMX in different samples including pharmaceutical formulations [12, 13, 16–20]. It is well-known that determination of biologically active compounds like drugs using bare electrodes is sometimes difficult due to its fauling property and high overpotential [3, 21]. Electrode surface modification changes the surface layers of the electrode itself or creates a layer with some form of chemical as well as physical selectivity [3, 20]. Electrodes have been modified using electroactive polymers and polymeric composites, metal complexes, alloys, and quantum dots among others [21–23].

Potentiodynamically fabricated poly(4-amino-3-hydroxynaphthalene sulfonic acid)-modified glassy carbon electrode (poly(AHNSA/GCE)), which has been sufficiently characterized in one of our previous work [24], is reported to exhibit catalytic properties towards selected electroactive analytes [24–27]. To the best of our knowledge, poly(AHNSA/GCE) has not been reported for electrochemical detection of amoxicillin in pharmaceutical tablet formulation. Hence, this work describes cyclic voltammetric investigation of the electrochemical behavior of amoxicillin, and standard addition square wave voltammetric determination of amoxicillin in four brands of tablet samples using poly(AHNSA)/GCE.

## Materials and methods

### Chemicals and apparatus

Amoxicillin (≥ 99.0%, Sigma Aldrich), K_3_[Fe(CN)_6_] and K_4_[Fe(CN)_6_] (98.0%, BDH laboratories supplies, England), potassium chloride (99.5%, Blulux laboratories (p) Ltd), sodium monohydrogen phosphate and sodium dihydrogen phosphate (≥ 98%, Blulux laboratories (p) Ltd), hydrochloric acid (37%, Fisher Scientific), sodium hydroxide (Extra pure, Lab Tech Chemicals), and 4-amino-3-hydroxynaphthalene-1-sulfonic acid (≥ 99.7%, Sigma Aldrich), all of analytical grade, were used without further purification.

CHI 760E potentiostat (Austin, Texas, USA), pH meter (AD8000, Romania), refrigerator (Lec refregration PLC, England), deionizer (Evoqua water technologies), and electronic balance (Nimbus, ADAM equipment, USA) were among the equipment/instruments used.

### Procedure

#### Preparation of poly(AHNSA) modified GCE

The poly(AHNSA) film coated glassy carbon electrode was prepared following reported procedure [24]. Briefly: the potential of a polished glassy carbon electrode in a 0.1 mol L^−1^ HNO_3_ containing 2.0 mmol L^−1^ 4-amino-3-hydroxynaphthalene-sulfonic acid (AHNSA) was scanned between − 0.8 and + 2.0 V for 15 cycles at a scan rate of 0.1 V s^−1^. The modified electrode, after rinsed with deionized water, was then stabilized in 0.5 mol L^−1^ H_2_SO_4_ by scanning the potential between − 0.8 and + 0.8 V until a steady cyclic voltammogram was obtained.

#### Preparation of standard amoxicillin solutions

5.0 mmol L^−1^ standard AMX stock solution in 100 mL volumetric flask was prepared by dissolving an accurately weighed amount of trihydrated amoxicillin in deionized water. Working standard AMX solutions were prepared from the stock solution by serial dilution with 0.1 mol L^−1^ phosphate buffer solution (PBS) of the required pH.

#### Preparation of tablet samples

Five weighed AMX tablets from each of the four studied tablet brands [Addis pharmaceutical factory (APF), Ethiopia; Ethiopian pharmaceuticals manufacturing factory (EPHARM), Ethiopia; Denk pharma GmbH (DENK), Germany; and Glaxo SmithKline Pharmaceuticals Ltd. (GLAXO), India], with average tablet mass of 602, 589, 675, and 608 mg/tablet, respectively were completely ground and homogenized using a mortar and pestle. Tablet sample stock solutions (1.91 mmol L^−1^ APF; 1.95 mmol L^−1^ EPHARM; 1.70 mmol L^−1^ DENK; and 1.89 mmol L^−1^ Glaxo) were prepared by transferring 42 mg powder from the respective tablet powder to a 50 mL volumetric flask and filled up to the mark with deionized water. Eight working tablet solutions with final AMX concentrations of 19.10 (APF), 19.50 (EPHARM), 17.02 (DENK), and 1.89 µmol L^−1^ in pH 5.5 PBS were further prepared from the respective intermediate solutions and spiked with standard AMX of various concentrations (1st–8th: 0, 10, 25, 50, 75, 100, 125, and 150 µmol L^−1^, respectively). The same procedure was followed for all the four brands of tablet samples.

### Electrochemical measurement

All electrochemical measurements were conducted using a conventional three electrode system with bare GCE or poly(AHNSA)/GCE as working electrode, Ag/AgCl (3.0 mol L^−1^ KCl) as reference electrode, and Pt coil as counter electrode. While cyclic voltammetry was used to evaluate the electrochemical behavior of AMX at the surface of the poly(AHNSA)/GCE there by study selected kinetic parameters, square wave voltammetry was employed for quantitative determination of amoxicillin in four brands of tablet formulations. All potentials in this experiment are against Ag/AgCl (3.0 mol L^−1^ KCl).

## Results and discussion

### Cyclic voltammetric study of AMX at poly(AHNSA)/GCE

#### Electrochemical behavior of AMX at poly(AHNSA)/GCE

The choice of poly(AHNSA) as a modifier in this study was from its reported improved conductivity, increased electrode surface area, and chemical and mechanical stability [24–27].

**Fig. 1:**
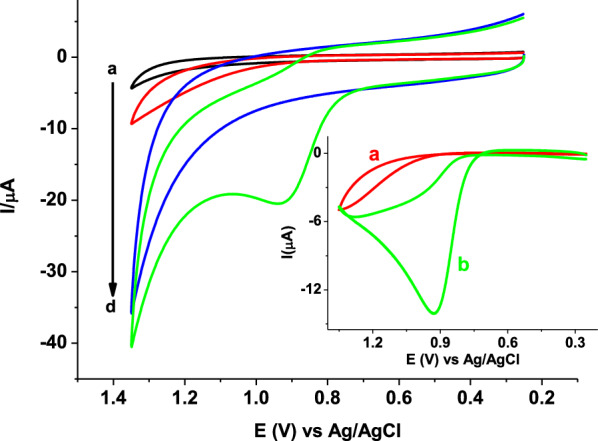
CVs of bare GCE (a, b) and poly(AHNSA)/GCE (c, d) in pH 7.0 PBS in the absence (a, c) and presence (b, d) of 1.0 mmol L^−1^ AMX at scan rate of 0.1 V s^−1^. Inset: corrected for blank CVs of (a) bare GCE, and (b) poly(AHNSA)/GCE

In order to verify applicability of the proposed modifier for AMX determination, cyclic voltammetric measurements of 1.0 mmol L^−1^ AMX in pH 7 PBS at bare GCE, and poly(AHNSA)/GCE were recorded under similar conditions (Fig. 1).

In contrast to the weak and broad oxidative peak centered at about + 1.2 V that appeared at the unmodified GCE (curve a of inset), a well-defined oxidative peak at about + 0.9 V with fourfold enhancement of oxidative current at poly(AHNSA)/GCE (curve b of inset) in the absence of a peak in the opposite scan direction indicated that AMX undergoes irreversible oxidation at both electrodes although with differing sensitivity. The observed catalytic effect of the modifier towards AMX oxidation explained by over potential reduction by about 370 mV, and four folds peak current enhancement might be accounted for the reported increased conductivity or surface area [24].

#### Effect of scan rate on the Ipa and Epa of AMX

**Fig. 2:**
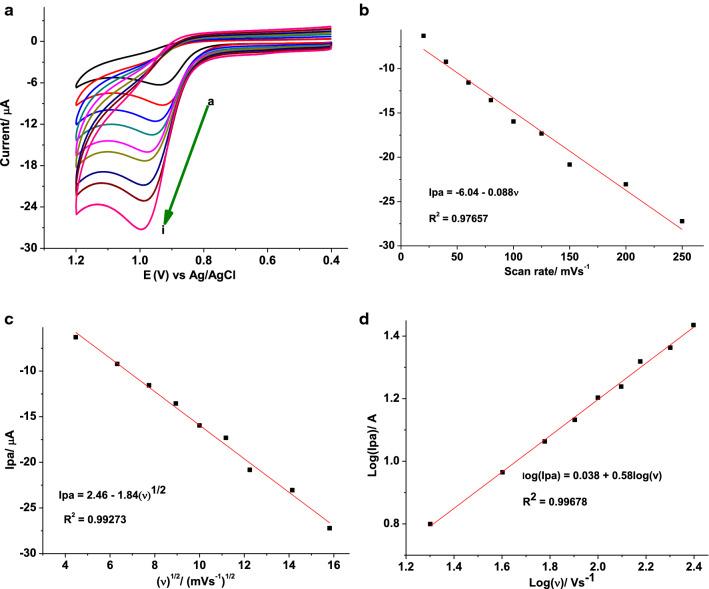
**a** CVs of poly(AHNSA)/GCE in pH 7.0 PBS containing 1.0 mmol L^−1^ AMX at various scan rates (a–i: 20, 40, 60, 80, 100, 125, 150, 200, and 250 mV s^−1^, respectively), (**b**) plot of Ip vs. $\nu$, (**c**) plot of Ip vs, $\nu^{1/2}$, and (**d**) plot of log (Ip) vs. log ($\nu$)

To confirm the irreversibility of the oxidation reaction and further investigate the rate determining step during oxidation of AMX at the modified electrode, the effect of scan rate on the peak potential and peak current was studied. Figure 2 presents voltammograms of 1.0 mmol L^−1^ AMX in pH 7.0 PBS at poly(AHNSA)/GCE in the scan rate range 20 to 250 mV s^−1^.

While the observed peak potential shift of AMX to a higher positive value with increasing the scan rate (Fig. 2a) confirmed the irreversibility of the oxidation of AMX at poly(AHNSA)/GCE, higher correlation (R^2^ = 0.9927) for the dependence of peak current on square root of scan rate (Fig. 2c) than on the scan rate (R^2^ = 0.9766) (Fig. 2b) indicated that the oxidation kinetics of AMX at the polymer modified electrode was predominantly controlled by mass transport [4]. Moreover, slope of 0.58 for plot of log (peak current) *versus* log (scan rate) (Fig. 2d), which is very close to the ideal 0.5 value for diffusion controlled, confirmed the diffusion mass transport kinetics of the oxidation of AMX at the polymer modified electrode [28].

The number of electrons involved in the oxidation of AMX at poly(AHNSA)/GCE was determined from cyclic voltammetric data. For an irreversible processes, the value of αn was determined by the difference between the peak potential (Ep) and the half-wave potential (E_1/2_), employing Eq. (1) [29]:

1 $$E_{p} - E_{1/2} = {\raise0.7ex\hbox{${47.7}$} \!\mathord{\left/ {\vphantom {{47.7} {\alpha n}}}\right.\kern-\nulldelimiterspace} \!\lower0.7ex\hbox{${\alpha n}$}},$$

where α is the arge transfer coefficient and n the number of electrons transferred.

Taking Ep and Ep_1/2_ from the cyclic voltammogram in the inset of Fig. 1 to be 928 and 839 mV, respectively, the value of αn from Eq. (1) was calculated to be 0.54. Considering α for totally irreversible electrode process to be 0.50 [30], the number of electrons (n) transferred in the electro-oxidation of AMX at the surface of poly(AHNSA)/GCE can be estimated as 1.08 (≈ 1.0).

The relationship between *Epa* and *lnν* of an irreversible process obeys the following Eq. (2) [23]:

2 $$Ep = E^\circ + \frac{RT}{{\left( {1 - \alpha } \right)nF}}\left\{ {0.780 + \ln \left( {\frac{{D_{R}^{\frac{1}{2}} }}{k^\circ }} \right) + \ln \left[ {\frac{{\left( {1 - \alpha } \right)nF\nu }}{RT}} \right]^{\frac{1}{2}} } \right\},$$

where *E*_*P*_ is the peak potential, *E*^*o*^ is the formal potential, α is the electron transfer coefficient, *k*^o^ (s^−1^) is the electrochemical rate constant, and the other parameters have their usual meanings.

**Fig. 3:**
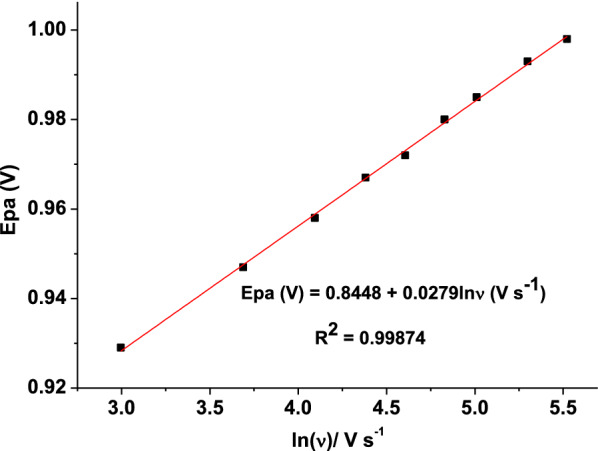
Plot of Ep vs. ln($\nu$)

From the slope (RT/(1 − α)nF = 0.0279) of the fitted line for plot of Ep versus ln $\nu$ (Fig. 3), the value of n(1 − α) at the experimental temperature of 25 °C was calculated to be 0.461. Taking the one electron oxidation of AMX found in Eq. (1), the electron transfer coefficient (α) was estimated to be 0.539 confirming the irreversibility of the reaction [30, 31].

#### Effect of supporting electrolyte on AMX at poly(AHNSA)/GCE

**Fig. 4:**
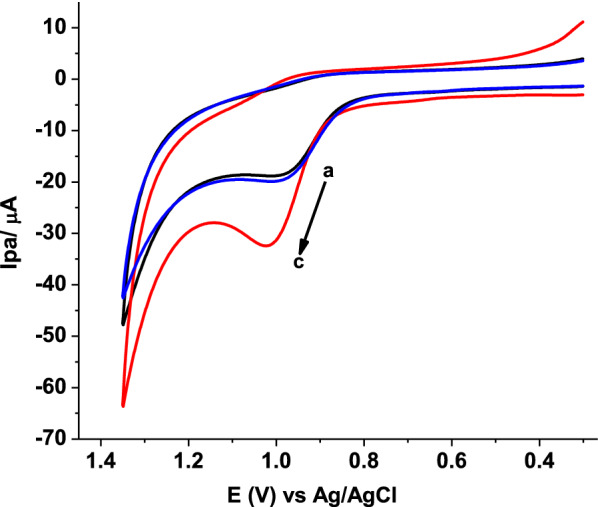
Cyclic voltammetric response of poly(AHNSA)/GCE for 1.0 mmol L^−1^ AMX in pH 5.0 of various electrolytes (a–c: ABS, RBS, and PBS, respectively)

The electrochemical behavior of an electroactive species usually depends on the type of supporting electrolyte and pH besides the nature of the electrode. The common types of supporting electrolytes; phosphate buffer solution (PBS), acetate buffer solution (ABS) and Britton-Robinson buffer solution (BRS), which all exhibit buffering capacity at pH 5.0 [18], were considered in this study. The voltammograms of 1.0 mmol L^−1^ AMX in pH 5.0 of the three buffer solutions at poly(AHNSA)/GCE are presented in Fig. 4. As can be observed from the figure, a well defined oxidative peak with an enhanced peak current for AMX in pH 5.0 PBS showed that the PBS is the best of the studied buffer solutions. Therefore, PBS prepared by mixing equi-molar (0.1 mol L^−1^) of NaH_2_PO_4_ and Na_2_HPO_4_ was used as a supporting electrolyte in this study.

#### Effect of pH on peak current and peak potential of AMX

**Fig. 5:**
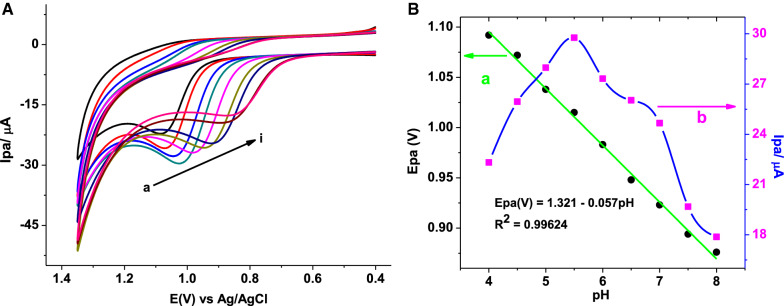
**A** CVs of poly(AHNSA)GCE in PBS of various pHs (a–i: 4.0, 4.5, 5.0, 5.5, 6.0, 6.5, 7.0, 7.5, and 8.0, respectively) containing 1.0 mmol L^−1^ AMX, **B** plot of (a) Ep and (b) Ip vs. pH in the entire pH range

Investigation of the effect of pH on the peak potential and peak current of an electroactive species at an electrode helps to investigate proton participation in a redox reaction, proton:electron ratio, and rationalize the possible type of interaction between the analyte and surface of the electrode. Cyclic voltammograms of poly(AHNSA)/GCE in PBS of various pHs containing 1.0 mmol L^−1^ AMX are shown in Fig. 5A. While observed peak potential shift in the negative direction with variation of pH from 4.0 to 8.0 (Fig. 4a) confirmed proton participation in the oxidation of AMX at the poly(AHNSA)/GCE, slope of 0.057 V for plot of oxidative peak potential versus pH of the supporting electrolyte (curve a for Fig. 5B) showed involvement of protons and electrons in a 1:1 ratio [32]. Moreover, the oxidative peak current of AMX at the surface of poly(AHNSA)/GCE is observed to increase with pH value from 4.0 to 5.5 which then decreased at pH values beyond it (curve b of Fig. 4b). AMX presents three pKa values at 2.6; 7.2 and 9.6. In acidic medium, it is protonated to give a cationic species. The increasing current trend in acidic region might be accounted partly for possible attraction between AMX (pKa 2.6; 7.2 and 9.6) with electrode modifier (pKa ≈ 4) [18, 27].

**Scheme 2:**
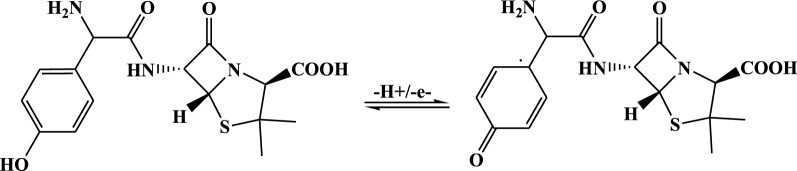
Proposed oxidation reaction mechanism of AMX

Based on the calculated number of protons and electrons participated in the oxidation of AMX at poly(AHNSA)GCE, a reaction mechanism (Scheme 2) which is in agreement with previously reported works is proposed [18].

### Square wave voltammetric investigation

**Fig. 6:**
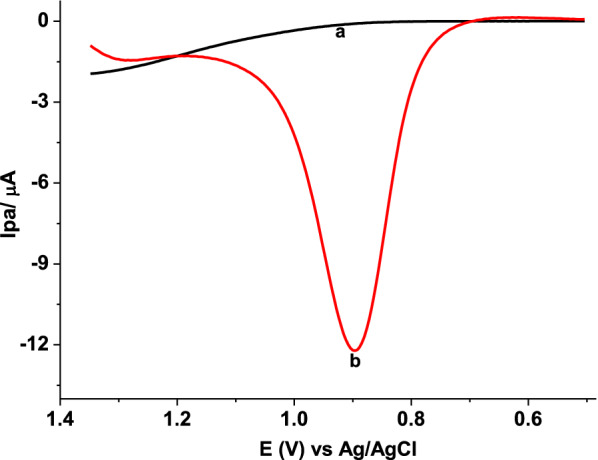
SWVs of (a) unmodified, and (b) poly(AHNSA)/GCE in pH 5.5 PBS containing 1.0 mmol L^−1^ AMX: (scan rate: 0.1 V s^−1^, step potential: 4 mV, amplitude: 25 mV, and frequency: 15 Hz)

Square wave voltammetry, which is more powerful to discriminate the Faradaic current from the non-Faradaic current than cyclic voltammetry [33], was used for quantification of AMX in tablet samples. Figure 6 presents square wave voltammograms (SWVs) of AMX in pH 5.5 PBS at bare GCE (a) and ploy(AHNSA)/GCE (b). In contrast to the peak at the unmodified electrode, appearance of a well-shaped square wave oxidative peak at poly(AHNSA)/GCE with about six folds of oxidative peak current at much reduced potential (≈ 400 mV) signified the catalytic contribution of the polymer film towards the oxidation of AMX.

For further analysis, the square wave voltammetric parameters such as step potential, pulse amplitude, and square wave frequency were optimized investigating the effect of each parameter keppeng the remaining constant. As expected, the oxidative peak current was increased with increasing every parameter although accompanied with peak broadening. As a compromise between the current enhancement and peak broadening, 8 mV, 35 mV, and 25 Hz were selected as the optimum step potential, amplitude, and frequency, respectively (Additional file 1: Figs. S1–S3).

### Determination of AMX in pharmaceutical tablet formulation

**Fig. 7:**
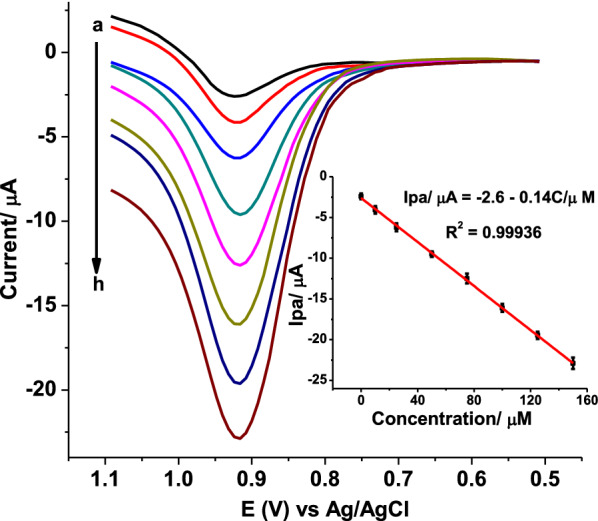
Background corrected SWVs of poly(AHNSA)/GCE in pH 5.5 PBS containing APF brand AMX tablet samples spiked with standard AMX (a–f: 0, 10, 25, 50, 75, 100, 125, and 150 µmol L^−1^, respectively) at optimized method parameters (E_step_ 8 mV, E_amp_ 35 mV, and frequency 25 Hz). Inset: plot of mean current (Ip ± SD, n = 3) vs concentration of spiked AMX

In this study, standard addition method of analysis was employed as a means to overcome the matrix effect in pharmaceutical tablet formulations. Figure 7 presents square wave voltammograms of APF brand AMX tablet sample spiked with various concentrations of standard AMX solutions as a representation of the four brands (APF, EPHARM, DENK, and GLAXO).

Under optimized conditions, the anodic peak current of the spiked tablet sample showed linear dependence on the spike concentration of standard AMX in the range 0.0 to 150 µmol L^−1^ for the studied four brands of tablets with *LOD* (*3 s/m, for n 7*) and *LOQ* (*10 s/m*) in the range of 9.93 × 10^–9^–1.02 × 10^–8^ mol L^−1^ and 3.31 × 10^–8^–3.41 × 10^–8^ mol L^−1^, respectively. Detection of AMX in an amount with adeviation from the claimed value by an amount below 3.1%, and %RSD under 6.58% (n = 3) showed the accuracy and precision of the developed method.

The detected amoxicillin content of the four tablet brands calculated using the respective regression equation, and the level as compared against the claimed AMX content are summarized in Table 1.

**Table 1:** Summary of standard addition regression equation, claimed AMX in the tablet sample, detected AMX content, and percent detected as compared to the theoretical value for each analysed tablet brand

Tablet brand	Linear regression equation; determination coefficient (R^2^)	Labeled AMX (mg/tablet)	claimed AMX in tablet sample (µmol L^−1^)	Detected AMX in		% detected
				Sample^a^ (µmol L^−1^)	Tablet (mg/tablet)	
APF	Ipa/µA = − 2.60 − 0.14C/µM; R^2^ = 0.99936	500	19.10	19.25 ± 5.82	503.90	100.78
EPHARM	Ipa/µA = − 2.50 − 0.13C/µM; R^2^ = 0.99929	500	19.50	19.08 ± 5.66	489.20	97.84
DENK	Ipa/µA = − 2.20 − 0.13C/µM; R^2^ = 0.99901	500	17.02	16.80 ± 6.07	493.50	98.70
GLAXO	Ipa/µA = − 2.50 − 0.13C/µM; R^2^ = 0.9995	500	18.90	18.70 ± 6.58	494.70	98.94

^a^Mean ± %RSD for n = 3

The AMX content of the four analysed tablet brands expressed as mg/tablet were determined and compared with the claimed value (500 mg/tablet). As can be seen from the table, the detected AMX content ranged between 97.84 (EPHARM) to 100.78% (APF) of the companies label. In contrast to the expected level of AMX in the four studied tablet brands, observed slight variations may ba accounted for experimental errors like possible mass loss during preparation, sort of degradation during storage, otherwise company error during preparation.

### Validation of the developed method

Besides the low LOD and LOQ, extremely small deviation of the detected level of AMX in the tablet samples compared to the claimed content, and low %RSD values for triplicate measurements which all validated the method, spike recovery and interference recovery studies were conducted to further validate the applicability of the developed standard addition method for determination of AMX in tablet formulation with complex matrix effect.

#### Recovery study

**Fig. 8:**
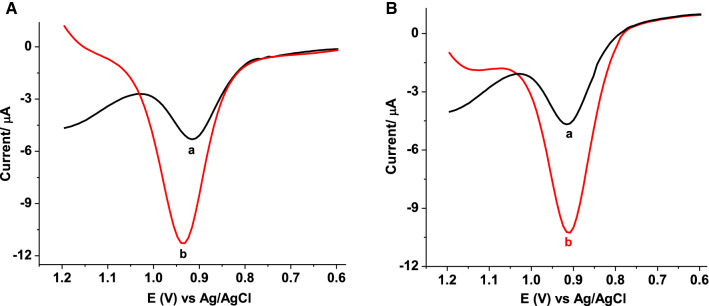
Corrected for blank corrent SWVs of poly(AHNSA)/GCE in pH 5.5 PBS containing **A** APF, and **B** EPHARM (a) unspiked tablet sample, and (b) a + 50 µmol L^−1^ standard AMX at optimized method parameters

APF and EPHARM tablet samples, which showed the highest and least AMX content among the studied four brands, were chosen for spike recovery studies. Two tablet solutions, both of 25 µmol L^−1^ AMX content for each brand, were prepared to one of which, 50 × 10^–6^ mol L^−1^ of standard AMX was spiked while the other remaining unspiked. Corrected for blank current square wave voltammograms of the unspiked (curve a) and spiked (curve b) APF (Fig. 8A) and EPHARM (Fig. 8B) are presented. As shown in Table 2, excellent recovery results of 100.5% for APF and 99.6% for EPHARM AMX tablet brands added to under 10% of %RSD (n = 3) validated applicability of the method for determination of AMX in tablet formulation.

**Table 2:** Summary of recovery results of 50 µmol L^−1^ AMX from tablet solutions containing 25 µM APF and EPHARM brand tablets

Tablet brand	Intial AMX content/(µmol L^−1^)	Spiked AMX (µmol L^−1^)	Detected AMX (µmol L^−1^)^a^	Recovery (%)
APF	25	–	25.08 ± 6.52	–
	25	50	75.33 ± 7.15	100.5
EPHARM	25	–	24.30 ± 5.78	–
	25	50	74.10 ± 6.12	99.6

^a^Mean ± %RSD for n = 3

#### Interference study

**Fig. 9:**
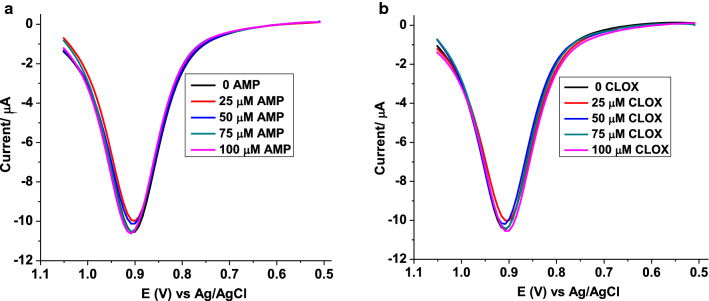
Corrected for blank SWVs of poly(AHNSA)/GCE in pH 5.5 PBS containing 50 µmol L^−1^ AMX tablet sample in the presence of **a** ampicillin, and **b** cloxicillin of various concentrations (0, 25, 50, 75, and 100 µmol L^−1^)

To further elaborate the potential applicability of the method, the selectivity of the method for AMX in the presence of selected potential interferents [ampicillin (AMP), and cloxicillin (CLOX)] was investigated. The selectivity of the method was studied by comparing the response of the method for 50 µmol L^−1^ AMX in the absence of the potential interfrents with the response recorded in their presence in the level of 50–200% of the AMX (Fig. 9a, b). Compared to the detected AMX in tablet sample in the absence of added interferent, detection of 95.4–100.8% AMX in the presence of AMP and CLOX (Table 3) validated the selectivity and specificity of the method for determination of AMX in samples with complex matrix.

**Table 3:** Summary of interference study of AMX with different concentrations of ampicillin, and cloxicillin

Interferent	Interferent added (µmol L^−1^)	Detected current (µA)^a^	Expected current (µA)	% recovery
AMP	0	10.48 ± 2.51	10.48	–
	25	10.00 ± 2.02	10.48	95.4
	50	10.20 ± 1.98	10.48	97.3
	75	10.48 ± 1.86	10.48	100.0
	100	10.57 ± 3.12	10.48	100.8
CLOX	0	10.48 ± 1.78	10.48	–
	25	10.10 ± 1.82	10.48	97.7
	50	10.24 ± 2.97	10.48	96.4
	75	10.42 ± 3.52	10.48	99.4
	100	10.52 ± 4.02	10.48	100.4

^a^Mean ± %RSD for n = 3

### Comparison with other methods

The performance of the present method was compared with selected recently reported voltammetric methods on the determination of amoxicillin in terms of the linear range, limit of detection, nature of the electrode substrate, and cost and availability of the electrode modifier.

As can be seen from Table 4, the present method based on poly(AHNSA)/GCE that requires simple electrode modification step, provides the least limit of detection, reasonably wider linear dynamic range than the others.

**Table 4:** Comparison of performance of the present method with selected recently reported works in terms of the linear dynamic range, LOD, electrode substrate, and modifier

Substrate	Modifier	Method	Dynamic range (µmol L^−1^)^a^	LOD (µmol L^−1^)	Refs.
GCE	QDs-P_6_LC-PEDOT:PSS	SWV	0.9–69.0	0.05	[12]
GCE	rGO/Nafion	SWV	1.8–5.4	0.36	[13]
CPE	Cu/POT(SDS)	CV	80–200	60	[14]
		Amperometry	5–150	3	
SPE	(AuNPs/en-MWCNT	CV	0.2–10; 10–30	0.015	[17]
Graphite electrode	TiO_2_/CMK/AuNPs/Nafion	CV	0.5–2.5; 2.5–133.0	0.3	[18]
GCE	Au NPs-Pd NPs-ErGO	SWV	30–350	9	[19]
GCE	CB–DHP	SWV	2.0–16.1	0.12	[20]
GCE	Poly(AHNSA)	SWV	10–150	0.0099	This work

^a^Mean ± %RSD for n = 3

Therefore, the reported method using relatively cheaper AHNSA modifier with the simplest modification procedure showed a comparable performance even with the methods that have used expensive electrode modifiers.

## Conclusion

In this work, the application of poly(AHNSA)/GCE,fabricated by electrodeposition of AHNSA film on the surface of glassy carbon electrode, for determination of AMX in four selected tablet brands is reported. Cyclic voltammetry was employed for the study of the electrochemical behavior of AMX, dependence of peak current on the pH of the solution and scan rate. In contrast to the cyclic voltammetric response recoded for AMX at unmodified glassy carbon electrode, appearance of an irreversible oxidation peak at reduced overpotential with sixfold current enhancement at Poly(AHNSA)/GCE signified excellent catalytic effect of the modifier towards AMX. Under optimized solution, and square wave voltammetric parameters, oxidative peak current response of the poly(AHNSA)/GCE showed linear dependence on the concentration of spiked standard AMX in a reasonably wide range of concentration. The amoxicillin content of the studied tablet samples determined using the present standard addition method ranged between 97.74 and 100.78% of their labels confirming the efficiency of the developed method. Wide dynamic concentration range, high precision, extremely low detection limit, excellent spike recovery results and high recovery results even in the presence of selected potential interferent validated the applicability of the developed method for determination of AMX in tablet samples, making the method an excellent potential candidate.

## Supplementary Information

**Additional file 1: Figure S1.** SWVs of poly(AHNSA)/GCE in pH 5.5 PBS containing 1.0 mol L^−1^ AMX at various step potential (a–d: 4, 8, 12, 16 mV, respectively), amplitude of 25 mV, and frequency of 15 Hz. Inset: Plot of Ip vs. step potential. **Figure S2.** SWVs of poly(AHNSA)/GCE in pH 5.5 PBS containing 1.0 mmol L^−1^ AMX at various square wave amplitudes (a–e: 25, 30, 35, 40, and 45 mV, respectively), step potential of 8 mV, and frequency of 25 Hz. Inset: Plot of Ip vs. amplitude. **Figure S3.** SWVs of poly(AHNSA)/GCE in pH 5.5 PBS containing 1.0 mmol L^−1^ AMX at step potential of 8 mV, amplitude of 35 mV, and various frequencies (a–f: 15, 20, 25, 30, 35, and 40 Hz, respectively). Inset: Plot of Ip vs. frequency.

## Data Availability

The dataset used and/or analysed in the current study are available from the corresponding author on request.
