# Supplementary material for: Poly(4-amino-3-hydroxynaphthalene-1-sulfonic acid) modified glassy carbon electrode for square wave voltammetric determination of amoxicillin in four tablet brands
Source: BMC Chem. 2021 Feb 8;15(1):10. doi: 10.1186/s13065-021-00739-0 (PMC7871396; doi:10.1186/s13065-021-00739-0)
Supplement: Supplementary file 1 — Additional file 1: Figure S1. SWVs of poly(AHNSA)/GCE in pH 5.5 PBS containing 1.0 mol L−1 AMX at various step potential (a–d: 4, 8, 12, 16 mV, respectively), amplitude of 25 mV, and frequency of 15 Hz. Inset: Plot of Ip vs. step potential. Figure S2. SWVs of poly(AHNSA)/GCE in pH 5.5 PBS containing 1.0 mmol L−1 AMX at various square wave amplitudes (a–e: 25, 30, 35, 40, and 45 mV, respectively), step potential of 8 mV, and frequency of 25 Hz. Inset: Plot of Ip vs. amplitude. Figure S3. SWVs of poly(AHNSA)/GCE in pH 5.5 PBS containing 1.0 mmol L−1 AMX at step potential of 8 mV, amplitude of 35 mV, and various frequencies (a–f: 15, 20, 25, 30, 35, and 40 Hz, respectively). Inset: Plot of Ip vs. frequency. [file 13065_2021_739_MOESM1_ESM.doc]

**Poly(4-amino-3-hydroxynaphthalene-1-sulfonic acid) modified glassy carbon electrode for square wave voltammetric determination of amoxicillin in four tablet brands**

**Adane Kassa^2^ and Meareg Amare^1^***

^1^Bahir dar University, Bahir Dar, Ethiopia

^2^Debremarkos University, Debremarkos, Ethiopia

*Corresponding author: [amaremeareg@yahoo.com](mailto:amaremeareg@yahoo.com)

**Additional file 1**

**Figure S1.** SWVs of poly(AHNSA)/GCE in pH 5.5 PBS containing 1.0 mol L^-1^ AMX at various step potential (a-d: 4, 8, 12, 16 mV, respectively), amplitude of 25 mV, and frequency of 15 Hz. Insert: Plot of Ip vs. step potential.

**Figure S2.** SWVs of poly(AHNSA)/GCE in pH 5.5 PBS containing 1.0 mmol L^-1^ AMX at various square wave amplitudes (a-e: 25, 30, 35, 40, and 45 mV, respectively), step potential of 8 mV, and frequency of 25 Hz. Insert: Plot of Ip vs. amplitude.

**Figure S3.** SWVs of poly(AHNSA)/GCE in pH 5.5 PBS containing 1.0 mmol L^-1^ AMX at step potential of 8 mV, amplitude of 35 mV, and various frequencies (a-f: 15, 20, 25, 30, 35, and 40 Hz, respectively). Insert: Plot of Ip vs. frequency**.**
